# Supplementary material for: Bipartite entanglement in a nuclear spin register mediated by a quasi-free electron spin
Source: Nat Commun. 2026 Mar 6;17:2325. doi: 10.1038/s41467-026-70154-3 (PMC12976098; doi:10.1038/s41467-026-70154-3)
Supplement: Supplementary file 1 — Supplementary Information [file 41467_2026_70154_MOESM1_ESM.pdf]

Supplementary Information: Bipartite entanglement in a nuclear spin register mediated by a quasi-free electron spin

Marco Klotz*[1], Andreas Tangemann*[1], David Opferkuch[1,2], and Alexander Kubanek†[1,2]

[1] *Institute for Quantum Optics, Ulm University, Albert-Einstein-Allee 11, 89081 Ulm, Germany*
[2] *Center for Integrated Quantum Science and Technology (IQST), Ulm University, Albert-Einstein-Allee 11, Ulm 89081, Germany*

01st December 2025

Supplementary Note 1: Additional electron spin characterization.

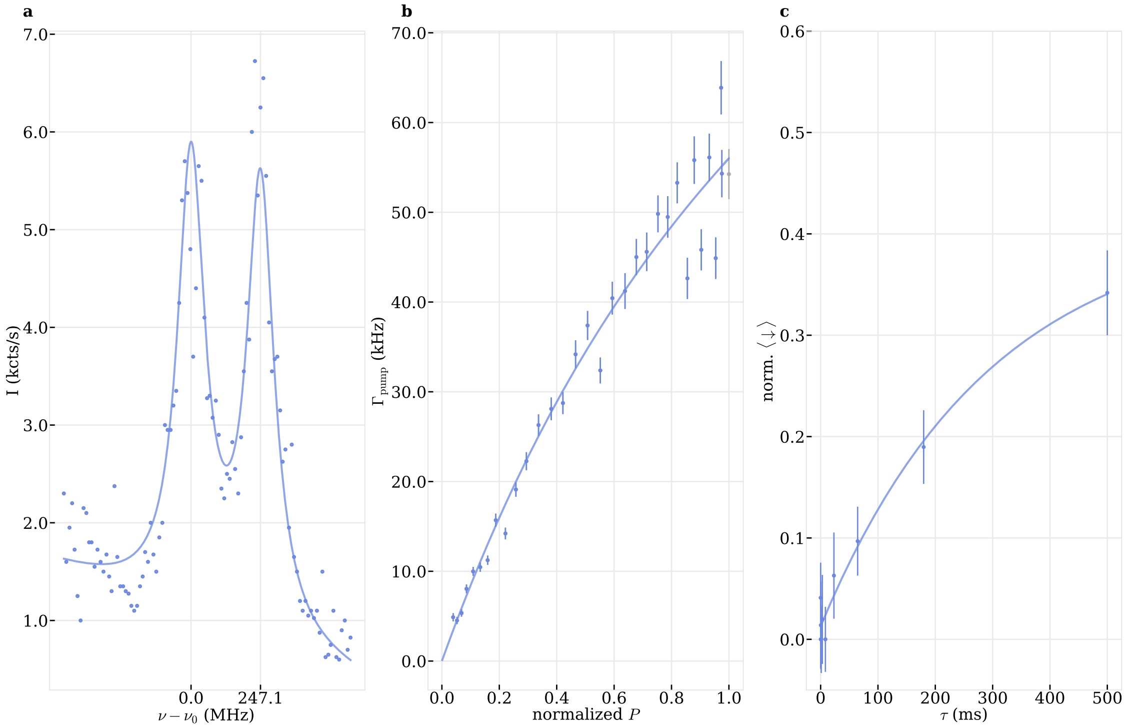

Supplementary Figure 1: Additional spin and optical properties a Photoluminescent excitation (PLE) scan where a low-power laser's frequency ν is scanned across the SiV's spin-split optical dipoles and the respective intensity I in the phonon-sideband is collected. **b** Power-dependent (P) spin pumping rate Γ_{pump} to extract the SiV's optical cyclicity. **c** Spin relaxation with a pump-probe measurement where pump laser-pulses are interleaved with variable wait times τ.

As described in [1] we biased the SiV prescreening towards highly strained SiVs by searching for a C-line at wavelengths above typical zero-strain wavelengths (approximately at 736.9 nm) and applying a continous-wave (CW) microwave field at the suspected Larmor frequency of a free electron to prevent spin pumping. In Supplementary Fig.1a the photo-luminescent excitation (PLE) scan of the SiV is depicted with a resonant CW-MW applied. The two spin-conserving transitions, fit with two Lorentzians with a linewidth of 117.1(72) MHz, are split by 247 MHz and centered around 737.871 776(13) nm. To extract the cyclicity η of the SiV, we measure power-dependent spin pumping rates Γ_{pump} by initializing the SiV electron spin with a resonant laser-pulse on one of the spin-cycling transitions, invert the spin state with a resonant microwave π-pulse and finally detect the fluorescence signal from the spin-pumping. We extract $\Gamma_{\text{pump}}(P)$ as a function of the laser power P from

an exponential fit to the fluorescence signal which is then fitted by the expression [2, 3]:

$$\Gamma_{\text{pump}} = \frac{\Gamma_0}{2}\frac{1}{\eta}\frac{s}{1+s}, \qquad s = P/P_{\text{sat}}. \tag{1}$$

where we used a typical excited-state lifetime $1/\Gamma_0 = 1.65\,\text{ns}$ [1]. The procedure resulted in $\eta = 2020(380)$. The increased cyclicity compared to a less strained SiV in [1] is inline with the higher strain and therefore stronger decoupling of the spin and orbital components of the SiV eigenstates. This also manifests itself in a significantly longer spin lifetime of $T_1 \approx 0.296(85)\,\text{s}$, see Supplementary Fig.1c, which is an increase of close to three orders of magnitude compared to a less strained SiV in[1].

Supplementary Note 2: Nuclear Spin Register Scalability

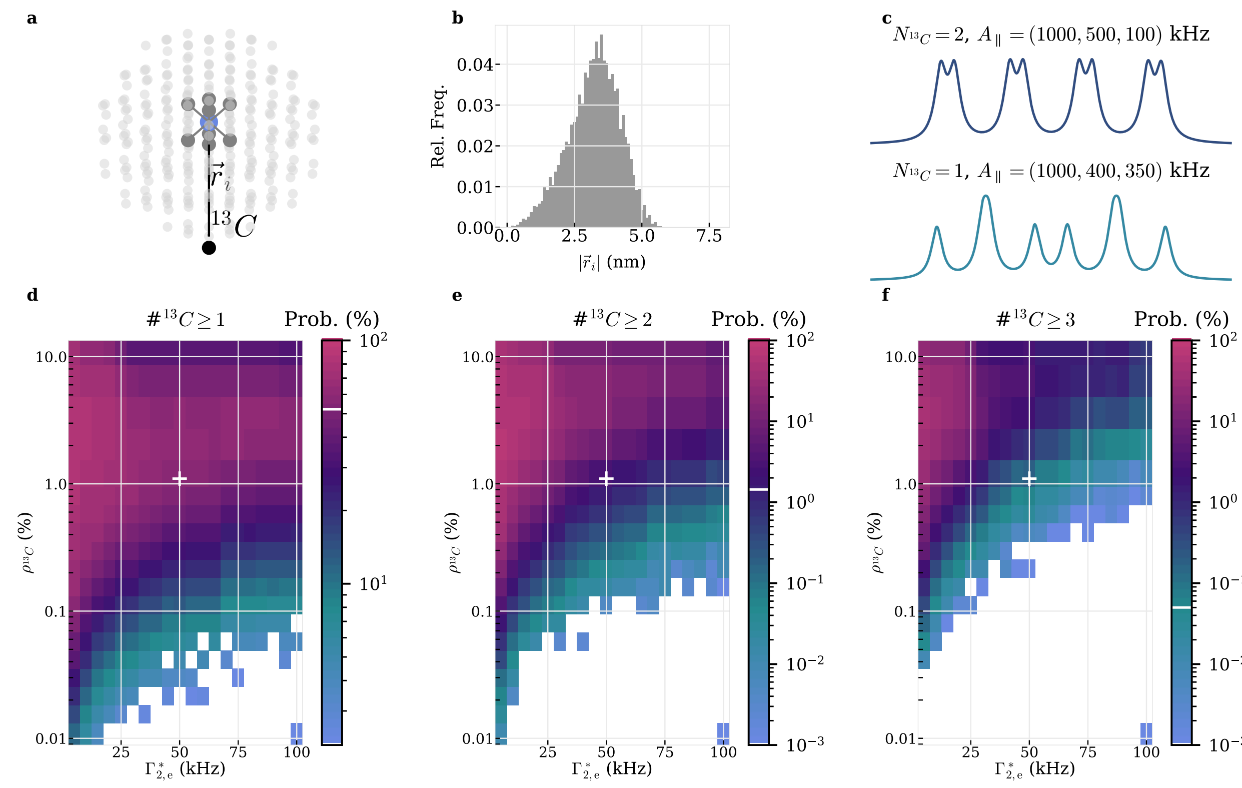

Supplementary Figure 2: a Artistic sketch of the diamond lattice (light grey), the SiV (blue) with its six nearest-neighbor carbon atoms (dark gey). The distance vector $\vec{r}_i$ to the i-th ^{13}C nuclear spin is indicated in black. **b** Histogram of the length of distance vectors $\vec{r}_i$ indicating a peak at around 3 nm. **c** Two examples of the classification procedure of usable nuclear spins. Top panel: two usable spins since $A_{\parallel}^{1} > A_{\parallel}^{2} + 2\Gamma_{2,\mathrm{e}}^{*}$ and $A_{\parallel}^{2} > A_{\parallel}^{3} + 2\Gamma_{2,\mathrm{e}}^{*}$. Bottom panel: only one usable spin, since $A_{\parallel}^{2} < A_{\parallel}^{3} + 2\Gamma_{2,\mathrm{e}}^{*}$ with $\Gamma_{2,\mathrm{e}}^{*} = 50$ kHz. **d - f** Probability of finding at least 1-3 usable nuclear spins.

The ^{13}C nuclear spin register we are using for entanglement generation is composed of three strongly coupled ($A_{\parallel}^{1-3} > \Gamma_{2,e}^{*}$) and an additional weakly coupled spin ($A_{\parallel}^{4} < \Gamma_{2,e}^{*}$). In order to explore the scalability of such a register, we perform Monte-Carlo simulations evaluate useful register configurations. To this end, we take a finite section of the diamond lattice with lattice positions $\vec{r}_i$, see Supplementary Fig.2a, and calculate the dipolar hyperfine tensor at each position

$$A^i/2\pi = h\,\frac{\mu_0}{4\pi}\gamma_e\,\gamma_{^{13}C}\,\frac{(3\,\hat{r}_i\otimes\hat{r}_i - \mathbb{I}_3)}{|\vec{r}_i|^3} \tag{2}$$

with unit distance vector $\hat{r}_i = \vec{r}_i/|\vec{r}_i|$ from each lattice to the SiV position, permeability of free space μ_0, the gyromagnetic ratio of the electron and nuclear spin $\gamma_e \approx 28.0\,\mathrm{GHz\,T^{-1}}$ and $\gamma_{^{13}C} \approx 10.7\,\mathrm{MHz\,T^{-1}}$ as well as the Planck constant h. In total we include ($\approx 5\cdot 10^4$) lattice positions such that the mean distance is roughly 3 nm, see Supplementary Fig.2b. We construct a set of $A_{\parallel}^{i}$ by looping through all lattice positions and include $A_{\parallel}^{i} = A_{zz}^{i}$ at each position with probability $\rho_{^{13}C}$. For a given set of $A_{\parallel}^{i}$ we then count the number of usable nuclear spins to form a register. We define two usability criteria in the following way:

1. We consider only those $\left|A_{\parallel}^{i}\right|$ which are larger than twice the dephasing rate $\Gamma_{2,e}^{*}$, i.e. $|A_{\parallel}^{1}|,\ldots,|A_{\parallel}^{N'}| > 2\Gamma_{2,\mathrm{e}}^{*}$
2. From that subset, we again loop through all $\left|A_{\parallel}^{i}\right|$ and classify the respective spin as usable if $|A_{\parallel}^{i}| > |A_{\parallel}^{i+1}|+2\,\Gamma_{2,\mathrm{e}}^{*}$, i.e its resonance is resolvable in ODMR from the next weaker coupled spin, see Supplementary Fig.2c.

In order to get enough statistic, we repeat this procedure 10^5 times and calculate a histogram s of the number of usable nuclear spins. From the histogram we determine a probability of finding at least N nuclear spins by $p(N) = \sum_{n=N}^{N_{\max}} s(n)/\sum_{n=0}^{N_{\max}} s(n)$, where $N_{\max}$ is the maximum number of spins found. Supplementary Fig.2d - f show the probability of finding at least 1 - 3 usable nuclear spins, where the white crosses indicate our

operation point at natural density $\rho_{^{13}C} = 1.1\%$ and the measured $\Gamma^*_{2,\mathrm{e}} \approx 50\,\mathrm{kHz}$ from the main text.
Under these conditions, we extract probabilities of a usable 1,2,3-qubit nuclear spin register of $52.10, 1.44, 0.05\%$, respectively. The Monte-Carlo simulation generally show that reducing $\Gamma^*_{2,\mathrm{e}}$ can drastically improve the number of usable nuclear spins. The improvement increases with increasing qubit number. For example, in the case of a three-qubit register it increases from $10^{-2}\%$ to $10^{0}\%$ when halving the electron spin's linewidth. This could be achieved by cooling the nuclear spin bath using global polarization techniques like NOVEL [4] or PulsePol[5], SWAP or projective measurements. Furthermore, the register size can be extended beyond strongly coupled spins by means of nuclear-nuclear couplings which can be characterized with SEDOR.

Supplementary Note 3: All-optical nuclear spin sensing.

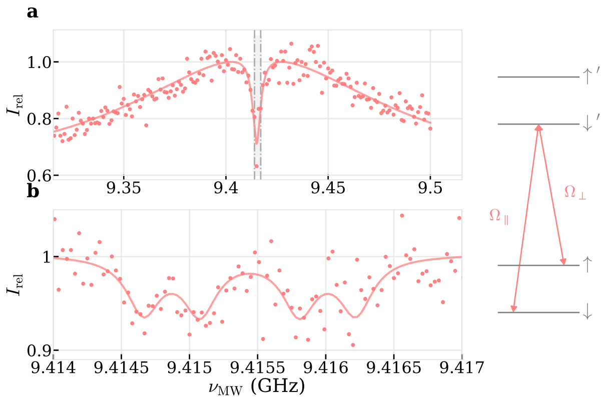

Supplementary Figure 3: Coherent population trapping a Resonant driving of the optical Λ-system consisting of the transitions $\nu_{\uparrow\downarrow'}$ and $\nu_{\downarrow\downarrow'}$. At the Raman condition, when both driving frequencies are resonant, the system is pumped into an optical dark state. Solid line is a double-Lorentzian fit. **b** By reducing the driving strength the hyperfine structure can be sensed optically using CPT, the spectrum is fit by the sum of four Lorentzians (solid line).

We resonantly drive the transition $\nu_{\uparrow\downarrow'}$ with $\Omega_\perp$ and scan another laser frequency, realized by the sideband of a locked electro-optical modulator, with $\Omega_\parallel$ across $\nu_{\downarrow\downarrow'}$ to match the Λ-system's Raman condition, i.e. $\nu_{\downarrow\downarrow'} - \nu_{\downarrow\uparrow} = \nu_{\text{L,e}}$, where $\nu_{\text{L,e}}$ is the electron spin's Larmor frequency.
The continuous driving of the Λ-system results in the preparation of a coherent dark state of the two involved optical dipoles which is decoupled from the driving lasers and hence results in a quench in fluorescence, see Supplementary Fig.3a, called coherent popoulation trapping (CPT).
Since the width of the dip is given by residual power-broadening and decoherence in the spin ground-states, we can estimate a decoherence rate $\Gamma_{2,e}^{*,\text{CPT}} = 149(24)\,\text{kHz}$ from the FWHM of a Lorentzian fit to a low-power measurement, Supplementary Fig.3b. We can resolve the strongest coupled nuclear spins all-optically and extract hyperfine coupling components $A_\parallel^1 = 1144(31)\,\text{kHz}$ and $A_\parallel^2 = 415(27)\,\text{kHz}$, inline with the measurements presented in the main text.
Additionally, the $\Gamma_{2,e}^{*,\text{CPT}}$ is close to the dephasing rate measured by Ramsey interferometry in the main text and the predicted dephasing rate using Monte Carlo simulations for an unpolarized nuclear spin bath.

Supplementary Note 4: Electron spin noise.

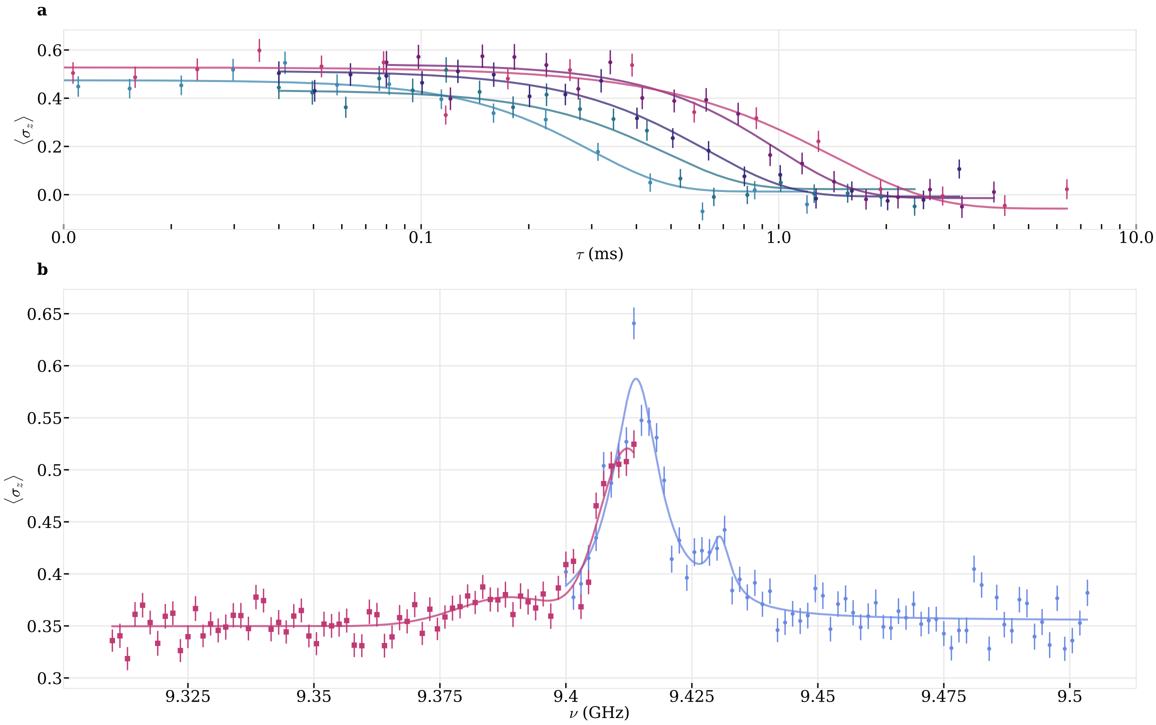

Supplementary Figure 4: Noise environment. **a** CPMG-N type dynamical decoupling with interpulse spacing τ which is used to fit and extract the respective $T_{2,\mathrm{e}}^{\mathrm{CPMG}}$ times from the main text. Solid lines are fits to $a\exp(-(\tau/T_{2,\mathrm{e}}^{\mathrm{CPMG}})^{\beta})+c$. **b** Double electron-electron resonance measurements. Here ν is the frequency of the probe π-pulse, trying to additionally invert target electron spins. Solid lines (red/blue) lines are each fits to the measured data with two Gaussian functions.

The data depicted in Supplementary Fig.4a are measurements, where we perform CPMG-type dynamical decoupling. For each data trace the number of decoupling pulses N is varied in the sequence $\frac{\pi_\mathrm{x}}{2} - [\frac{\tau}{2} - \pi_\mathrm{y} - \frac{\tau}{2}]^N - (\frac{\pi_{\pm\mathrm{x}}}{2})$, where τ is the free evolution time and $\pi_{\mathrm{x,y}}$ indicate orthogonal rotations of the electron spin on the Bloch sphere. The last $\pi/2$-pulse is performed around the $\pm$x-axis to read out the electron spin contrast σ_z. We extract the coherence times $T_{2,\mathrm{e}}^{\mathrm{CPMG}}$ from stretched exponential functions with stretching factor β in the range of $1.8-4$. The coherence times scale with $T_{2,\mathrm{e}}^{\mathrm{CPMG}} \propto N^{\chi}$, $\chi = 0.513(26)$ diverting from the expected scaling of $\chi = 2/3$ for a nuclear spin bath [6–8]. This behavior can be explained by coupling to additional noise sources such as free electrons [9–11]. To further investigate the noise bath we perform double electron-electron resonance (DEER) spectroscopy by performing a Hahn echo with interpulse spacing τ on our SiV electron spin and synchronously to the rephasing π-pulse apply an additional π-pulse with frequency ν, thereby trying to recouple potential target spins.

In Supplementary Fig.4b we use a free precession time $\tau = 100\,\mu\mathrm{s}$ and probe potential spin frequencies below/above (red/blue) the SiV electron spin resonance frequency of 9.414 GHz. We measure a broad resonance at $\nu_{\mathrm{bath},1} = 9.3889(38)$ GHz with FWHM $\Gamma_{\mathrm{DEER},1} = 24.4(95)$ MHz, extracted from a Gaussian fit. This agrees well with the expected Larmor frequency of free electrons in the static magnetic field of 335 mT.

Above the resonance of the SiV we measure a second resonance with a loss-of-coherence at $\nu_{\mathrm{DEER},2} = 9.430\,64(77)$ GHz with a $\Gamma_{\mathrm{DEER},2} = 5.0(25)$ MHz which could indicate coupling to the electron spin of a different, nearby defect center.

Supplementary Note 5: Dynamically decoupled nuclear spin initialization

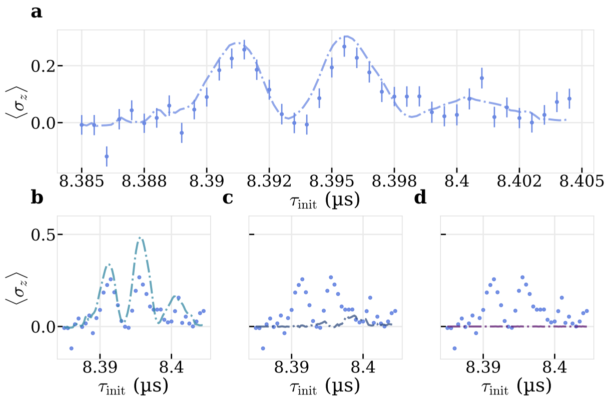

Supplementary Figure 5: Dynamically decoupled nuclear spin initialization. **a** Sweeping τ_{init} of the DD initialization sequence from the main text Fig.3d. The electron spin's contrast σ_{z} indicates successful population transfer for different. The dash dotted line is extracted from the numerical model, see Supplementary Note 6. **b-d** The simulations (dash-dotted lines) are showing the population transfer for individual n_{1-3}, respectively. The measured contrast (dots) can be explained by population transfer to n_1.

In Supplementary Fig.5a the measurement data from the pulse sequence depicted in Fig.3d to initialize nuclear spins using dynamical decoupling on the electron spin is shown. We sweep the inter-pulse delay τ_{init} of each rotation block $R_{\pm}(\tau, N)$ around the resonances, see Fig.3b in the main text, and use $N = 24$ corresponding to a conditional $\pi/2$ rotation of n_1. Afterwards, we reverse the sequence to transfer the nuclear spin's population back on the electron spin followed by laser readout. The measured electron spin's contrast σ_{z} indicates the successful population transfer. The dash-dotted line displays the expected contrast calculated with the numerical model with the three register nuclear spins and the additional fourth nuclear spin, parameters as in Supplementary Table 1. In Supplementary Fig.5b-c the same data as in Fig.5a is presented (dots) as a reference together with dash-dotted lines showing the simulated population transfer if only n_{1-3}, respectively, are taken into account in the model. From the simulations we conclude that the main contribution to the measured contrast shown in Fig.5a is from n_1. The increasing discrepancy to the model when increasing τ_{init} indicates that the gate fidelity is reducing due to the increasing contributions of the other nuclear spins n_{1-2}. For the chosen $\tau_{\text{init}} = 8.3915\,\mu\text{s}$ for the Ramsey measurements depicted in the main paper Fig.3e, the nuclear spins n_2(c) and n_3(e) are not polarized.

Supplementary Note 6: Low-Power Rabi measurements.

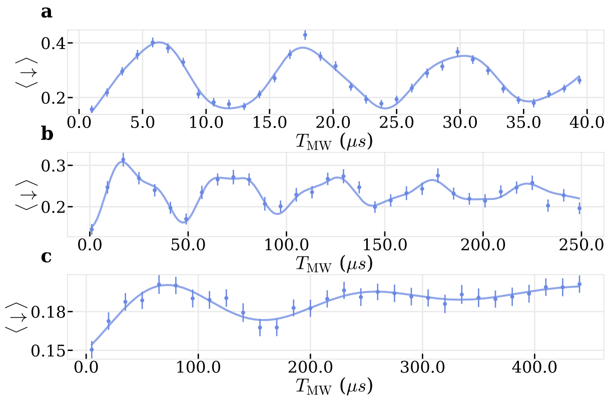

Supplementary Figure 6: Low-power Rabi measurements. **a** Beat of n_2 and n_3 with frequencies $\Omega_{\mathrm{R,e}}^{\mathrm{eff,1}}/2\pi = 83.47(50)\,\mathrm{kHz}$ and $\Omega_{\mathrm{R,e}}^{\mathrm{eff,2}}/2\pi = 201.8(53)\,\mathrm{kHz}$. **b** Beat of n_3 and n_3 with $\Omega_{\mathrm{R,e}}^{\mathrm{eff,1}}/2\pi = 19.50(25)\,\mathrm{kHz}$ and $\Omega_{\mathrm{R,e}}^{\mathrm{eff,2}}/2\pi = 43.0(14)\,\mathrm{kHz}$. **c** Ultra-lower power electron spin Rabi oscillations.

We use the ability to perform low-power Rabi oscillations on the electron spin to probe the nuclear spin spectrum in the time-domain owing to the long coherence under continuous driving. Supplementary Fig.6a shows a beat in a Rabi measurement, where we first polarize the two strongest nuclear spins and drive Rabi oscillations on the lowest energy electron spin transition. From a fit to exponentially damped harmonics, we extract two dominant effective Rabi frequencies of $\Omega_{\mathrm{R,e}}^{\mathrm{eff,1}}/2\pi = 83.47(50)\,\mathrm{kHz}$ and $\Omega_{\mathrm{R,e}}^{\mathrm{eff,2}}/2\pi = 201.8(53)\,\mathrm{kHz}$, respectively. We extract an effective detuning $\Delta/2\pi = \sqrt{\Omega_{\mathrm{R,e}}^{\mathrm{eff,2}} - \Omega_{\mathrm{R,e}}^{\mathrm{eff,1}}}/2\pi = 183.7(58)\,\mathrm{kHz}$. The discrepancy compared to the $A_{\parallel}^{3} = 144.6(71)$, determined in the main text, can be explained by a detuning of both microwave frequencies, for example due to coupling to additional nuclear spins.

The same procedure can be repeated by further decreasing the MW-power, see Supplementary Fig.6b. Here, we again observe a beating with $\Omega_{\mathrm{R,e}}^{\mathrm{eff,1}}/2\pi = 19.50(25)\,\mathrm{kHz}$ and $\Omega_{\mathrm{R,e}}^{\mathrm{eff,2}}/2\pi = 43.0(14)\,\mathrm{kHz}$, from which we extract $\Delta/2\pi = 38.65(85)\,\mathrm{kHz}$.

We are capable of measuring Rabi frequencies down to $\Omega_{\mathrm{R,e}}^{\mathrm{eff}}/2\pi = 5.49(22)\,\mathrm{kHz}$ with a coherence time of $T_{2,\mathrm{e}}^{\mathrm{Rabi}} = 161(30)\,\mu\mathrm{s}$, see Supplementary Fig.6c.

Supplementary Note 7: Sinc-pulse fidelity.

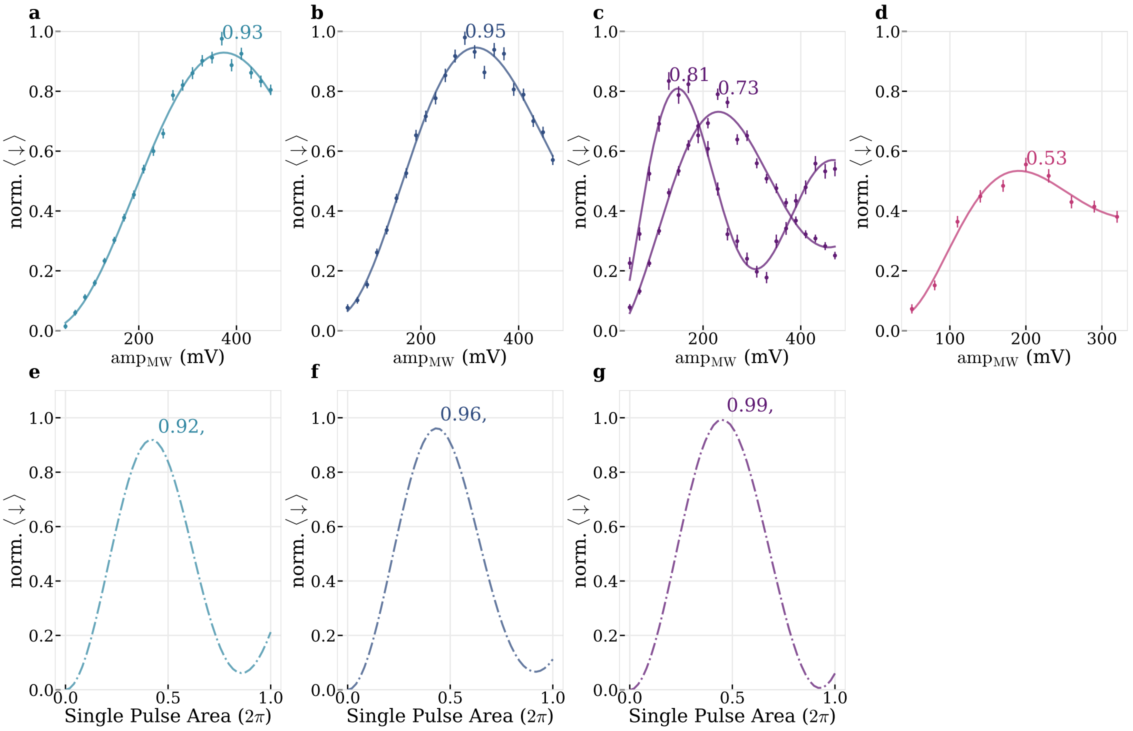

Supplementary Figure 7: Amplitude sweep of sinc-shaped pulses. In order to find optimal electron spin inversion, we sweep the AWG's MW amplitude $\mathrm{amp_{MW}}$ of a truncated sinc-shaped pulse with bandwidths **a** 1200 kHz, **b** 500 kHz, **c** 150 kHz and **d** 40 kHz with additional variable attenuation levels of a digital step attenuator. From an exponentially damped sine (solid line), we extract the respective gate-fidelities. For the case of 150 kHz bandwidth(c), we added a measurement with less MW attenuation to drive a 2π pulse, used for entanglement generation in the main text. **e-g** Simulation of the sinc-shaped pulse-amplitude sweep using the numerical model for bandwidths **e** 1200 kHz, **f** 500 kHz, **g** 150 kHz We normalize the amplitudes to the whole pulse area of a sinc-pulse.

In order to drive nuclear spin conditioned electron spin transitions, we use temporally sinc-shaped pulses (rectangularly-shaped in the frequency domain) truncated at the second zero-crossing to reduce temporal extent. The bandwidth B of the pulses are chosen to be approximately $A_{\|}^{i}/2\pi$. In order to realize high and low-bandwidth pulses within one pulse sequence, for example for the strong reference π-pulse at the beginning of each sequence, we utilize a digital step attenuator. The latter is controlled in real-time by an attenuation-level encoded pulse from the AWG on the order of 1µs sent to a FPGA which sets the respective level. Supplementary Fig.7a-d show the respective measurement data, where we apply the shaped pulses on all 2^{N_spins} resonances sequentially, where N_spins is the number of involved nuclear spins, given by the bandwidth of the pulses. For example, when $B = 1200$ kHz, we only use the 2^1 resonances at $(\omega_\mathrm{L,e} \pm A_{\|}^{1}/2)/2\pi$, since $B \gg A_{\|}^{2-4}$. From a fit of an exponentially damped sine we then extract the gate-fidelities.
To understand the infidelities, we reproduce the measured data with the numerical model, displayed in Supplementary Fig.7e-g. Since the AWG's output voltage is not calibrated to the experiment, we normalize the amplitudes to the pulse area such that the simulated Rabi frequency becomes $\Omega_\mathrm{R,e}(t)/2\pi = \frac{a}{\int_0^T \mathrm{sinc_{trunc}}(t)dt}\mathrm{sinc_{trunc}}(t)$, where $\mathrm{sinc_{trunc}}(t)$ is the truncated sinc-pulse and a is the swept amplitude. We then add rotating frames during application of the respective $\mathrm{C_{n_\mathit{i}}NOT}_e$ frequencies to the system's Hamiltonian (see Methods) and let the system evolve for a time $T \cdot 2^{N_\mathrm{spins}}$ under driving after which we evaluate the electron spins expectation value and normalize it by $(\langle\downarrow\rangle - (1 - F_\mathrm{e}))/(2F_\mathrm{e} - 1)$ resulting in Supplementary Fig.7e-g.
Comparing the simulation for the 1200 kHz and 500 kHz pulse with the respective measurements shows good agreement and validates the numerical model. However, comparison of the 150 kHz pulse shows a large discrepancy which we attribute to a combination of detuning in the measurement due to additional nuclear spins not taken into account in the system Hamiltonian ($N_\mathrm{spins} = 4$) and onset of decoherence during the sinc-pulses 26.6 µs. In addition, one can observe that the maximum population inversion does not happen at a normalized amplitude of $0.5 \cdot 2\pi = \pi$ but earlier, reflecting a fast over-rotation due to cross-talk. For the RF nuclear spin driving simulations and experiment in the main text we used the amplitudes of maximum inversion.

Supplementary Note 8: Nuclear Rabi spectroscopy

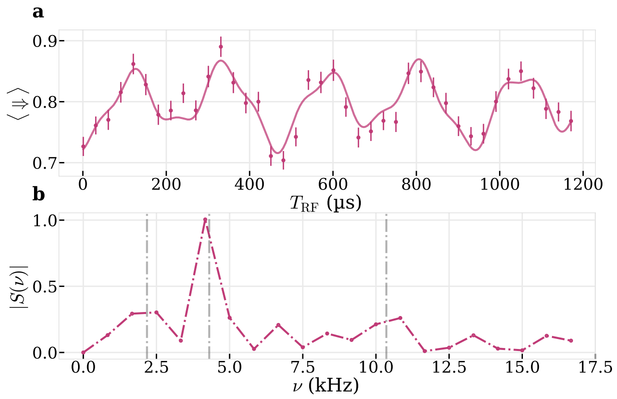

Supplementary Figure 8: Nuclear Rabi spectroscopy. a Nuclear Rabi oscillations on n_4 showing multiple frequency components, indicating an additional spin. bf Corresponding Fourier transform with fitted frequency components from **a** (grey dash-dotted lines).

We attempt to control the fourth nuclear spin n_4, barely resolvable in the electron ODMR spectrum presented in Fig.4c of the main manuscript by initializing with two CNOTs and then performing a Rabi experiment on the nuclear spin. When driving the nuclear spin n_i with the same RF-amplitude as the other nuclear spins, we observe additional harmonics, see Fig.8a. We extract three frequency contributions, highlighted in Supplementary Fig.8b by dashed lines. The main frequency component $\Omega^{0}_{\mathrm{R,n_4}}/2\pi = 4.304(42)\,\mathrm{kHz}$ is the expected resonant Rabi frequency. The lower frequency component $\Omega^{\mathrm{eff},1}_{\mathrm{R,n_4}}/2\pi = 2.18(12)\,\mathrm{kHz}$ is a non-physical measurement artifact. We attribute the higher frequency component $\Omega^{\mathrm{eff},2}_{\mathrm{R,n_4}}/2\pi = 10.36(15)\,\mathrm{kHz}$ to off-resonant driving of a fifth nuclear spin, that is also partially polarized due to a low gate selectivity of the CNOTs used to target n_4. From the effective Rabi frequency $\Omega^{\mathrm{eff},2}_{\mathrm{R,n_4}} = \sqrt{(\Omega^{\mathrm{eff},0}_{\mathrm{R,n_4}})^2 + \Delta^2}$ we extract a detuning of the applied RF drive of $\Delta/2\pi \approx 9.4\,\mathrm{kHz}$ and a potential $A^{5}_{\parallel}/2\pi \approx 18.7\,\mathrm{kHz}$. By reducing the RF driving strength resonant with n_4, the beat vanishes, see Fig.5d of the main text, further substantiating the origin of the beat hinting to at least one additional fifth nuclear spin.

Supplementary Note 9: Register post-selection.

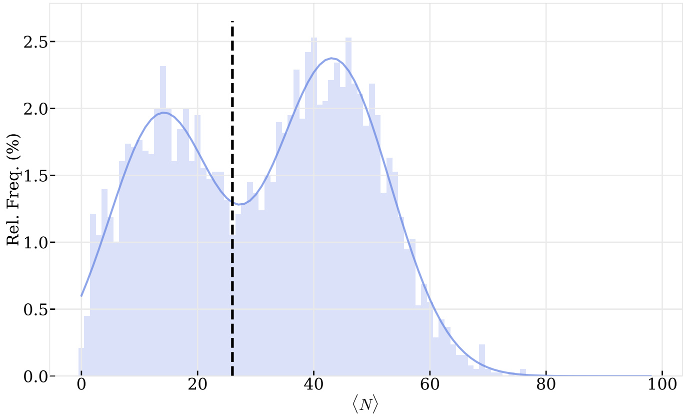

Supplementary Figure 9: Register post-selection. Photon statistics of $|\Uparrow_1\Uparrow_2\Uparrow_3\rangle$, with a double-Gaussian fit to extract a threshold number of photons, black dashed line, which optimizes dark and bright state's mutual overlap.

In order to increase the register's initialization fidelity of $|\Uparrow_1\Uparrow_2\Uparrow_3\rangle$ after performing single-shot readout (SSR) on each nuclear spin n_i, we additionally apply a SSR with a π-pulse conditional on $|\Uparrow_1\Uparrow_2\Uparrow_3\rangle$. The SSR parameters are given in the main text.
Supplementary Fig.9 shows the respective detected photon number distribution, where we discriminate a dark and bright state with a fidelity of 0.94, which we extracted from a double-Gaussian fit and a subsequent optimized threshold, indicated with a dashed line.

Supplementary Note 10: Additional Bell-states

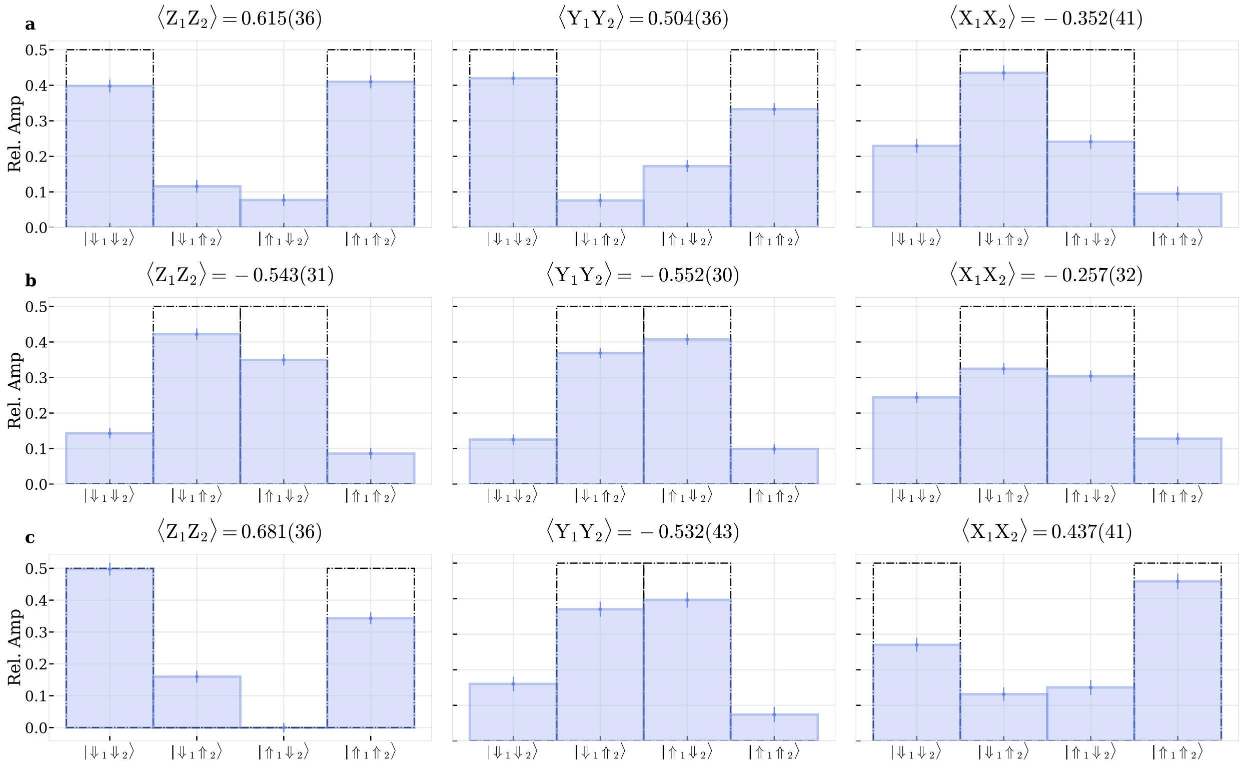

Supplementary Figure 10: Additional Bell-states.a-c By choosing different resonances for the CPhase gate, the three Bell-states (a) $\{|\Downarrow_1\Downarrow_2\rangle - |\Uparrow_1\Uparrow_2\rangle$, (b) $|\Downarrow_1\Uparrow_2\rangle - |\Uparrow_1\Downarrow_2\rangle$, (c) $|\Downarrow_1\Downarrow_2\rangle + |\Uparrow_1\Uparrow_2\rangle\}$ (neglecting normalization) not shown in the main article can be prepared with fidelities $F = \{$ (a) 0.618(16),(b) 0.588(13), (c) 0.663(17)$\}$.

By using the same sequence as depicted in the main paper Fig.7b, but conditioning the C-Phase gate on the states $\{|\Downarrow_1\Uparrow_2\rangle, |\Uparrow_1, \Downarrow_2\rangle, |\Uparrow_1, \Uparrow_2\rangle\}$, the remaining three (unnormalized) Bell states (a) $\{|\Downarrow_1\Downarrow_2\rangle - |\Uparrow_1\Uparrow_2\rangle$, (b) $|\Downarrow_1\Uparrow_2\rangle - |\Uparrow_1\Downarrow_2\rangle$, (c) $|\Downarrow_1\Downarrow_2\rangle + |\Uparrow_1\Uparrow_2\rangle\}$, are prepared. As reported in the main paper, the fidelity F of the Bell-states is evaluated by extracting the amplitudes for the three measured correlators together with appropriate coefficients c_i, depending on the Bell-state, as $F = \{$(a) 0.618(16),(b) 0.588(13), (c) 0.663(17)$\}$, see Supplementary Fig.10.
The fidelity of the Bell states in Supplementary Fig.10 (a) and (b) is reduced compared to (c) and main Fig.7c ($|\Downarrow_1\Uparrow_2\rangle + |\Uparrow_1\Downarrow_2\rangle$), because the electron spin resonances used for the CPhase gates, i.e. $|\Downarrow_1\Uparrow_2\rangle$, $|\Uparrow_1\Downarrow_2\rangle$, have both neighboring lower- and higher-frequency resonances, as opposed to $|\Downarrow_1\Downarrow_2\rangle$ and $|\Uparrow_1\Uparrow_2\rangle$. Due to the limited bandwidth of the truncated sinc-pulses used for the CPhase gate, these are partially addressed as well, reducing the Bell-state preparation fidelity.

References

1. Klotz, M., Tangemann, A. & Kubanek, A. Ultra-high strained diamond spin register with coherent optical control. *npj Quantum Information* **11,** 91 (2025).
2. Klotz, M. *et al.* Prolonged Orbital Relaxation by Locally Modified Phonon Density of States for the Si V-Center in Nanodiamonds. *Physical Review Letters* **128,** 153602 (2022).
3. Rosenthal, E. I. *et al.* Single-shot readout and weak measurement of a tin-vacancy qubit in diamond. *Physical Review X* **14,** 041008 (2024).
4. Henstra, A., Dirksen, P., Schmidt, J. & Wenckebach, W. T. Nuclear spin orientation via electron spin locking (NOVEL). *Journal of Magnetic Resonance (1969)* **77,** 389–393 (1988).
5. Schwartz, I. *et al.* Robust optical polarization of nuclear spin baths using Hamiltonian engineering of nitrogen-vacancy center quantum dynamics. *Science advances* **4,** eaat8978 (2018).
6. Medford, J. *et al.* Scaling of dynamical decoupling for spin qubits. *Physical review letters* **108,** 086802 (2012).
7. De Lange, G., Wang, Z., Riste, D., Dobrovitski, V. & Hanson, R. Universal dynamical decoupling of a single solid-state spin from a spin bath. *Science* **330,** 60–63 (2010).
8. Myers, B. A. *et al.* Probing surface noise with depth-calibrated spins in diamond. *Physical Review Letters* **113,** 027602 (2014).
9. Nguyen, C. T. *et al.* An integrated nanophotonic quantum register based on silicon-vacancy spins in diamond. *Phys. Rev. B* **100,** 165428. `https://link.aps.org/doi/10.1103/PhysRevB.100.165428` (16 Oct. 2019).
10. Beukers, H. K. *et al.* Control of solid-state nuclear spin qubits using an electron spin-1/2. *Physical Review X* **15,** 021011 (2025).
11. Rosenthal, E. I. *et al.* Microwave spin control of a tin-vacancy qubit in diamond. *Physical Review X* **13,** 031022 (2023).
